# Pharmacokinetic study of traditional Japanese Kampo medicine shimotsuto used to treat gynecological diseases in rats

**DOI:** 10.1007/s11418-020-01474-x

**Published:** 2021-01-04

**Authors:** Mikina Takiyama, Takashi Matsumoto, Sho Sanechika, Junko Watanabe

**Affiliations:** Tsumura Kampo Research Laboratories, Kampo Research and Development Division, Tsumura & Co., 3586 Yoshiwara, Ami-machi, Inashiki-gun, Ibaraki, 300-1192 Japan

**Keywords:** Shimotsuto, Active ingredients, Pharmacokinetics, Gynecological disease, Blood and circulatory system

## Abstract

**Abstract:**

Shimotsuto is a traditional Japanese Kampo medicine used to treat gynecological diseases, such as irregular menstruation, in addition to oversensitivity to cold and chilblains. Part of the pharmacological actions of shimotsuto is traditionally considered to be exerted by an improvement effect of the blood and the circulatory system. Multiple ingredients (e.g., catalpol and paeoniflorin) contained in shimotsuto have been reported to have pharmacological activities on the blood and circulatory system, and thus been considered to contribute to the pharmacological actions of shimotsuto. However, it remains unclear whether the ingredients can be absorbed into the body following oral administration of shimotsuto. The aim in the present study was to specify shimotsuto ingredient absorbed into the systemic circulation in rats. Seven candidate active ingredients (catalpol, paeoniflorin, albiflorin, ligustilide, senkyunolide A, butylphthalide, and ferulic acid) in plasma after oral administration of shimotsuto were quantified by targeted liquid chromatography–tandem mass spectrometry (LC–MS/MS) analysis. This study also performed nontargeted LC–MS/MS analysis of plasma following administration of constituent crude drugs of shimotsuto to find extensively blood-absorbed ingredients of shimotsuto. Among detected peaks in the nontargeted analysis, two peaks could be identified as bergapten and 8-debenzoylpaeoniflorin, subsequently their concentrations in shimotsuto-treated rat plasma were quantified. These pharmacokinetic studies indicated that catalpol showed the highest plasma concentration following administration of shimotsuto, followed by 8-debenzoylpaeoniflorin. This study suggests that all nine ingredients are absorbed into the blood following oral administration of shimotsuto and possibly contribute to its pharmacological action.

**Graphic abstract:**

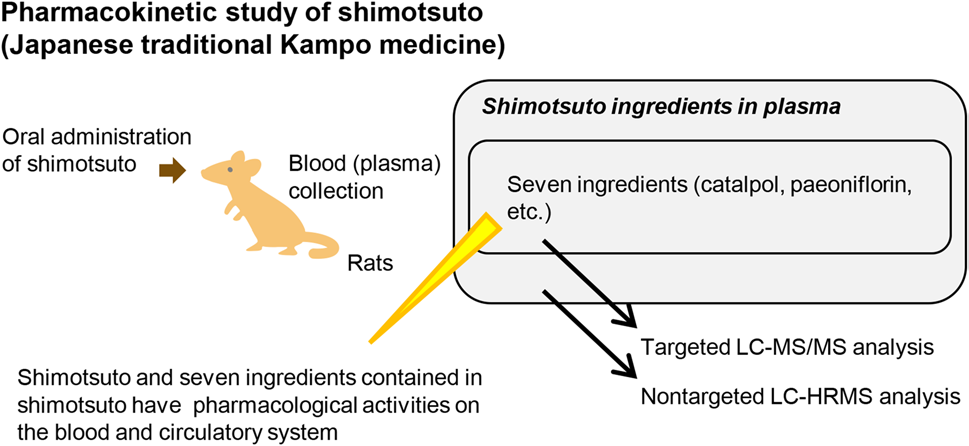

**Supplementary Information:**

The online version of this article (10.1007/s11418-020-01474-x) contains supplementary material, which is available to authorized users.

## Introduction

Traditionally, some herbal medicines have been used to treat gynecological diseases, especially in Asia [1–3]. The pharmacological actions of these medicines are believed to be due to their multiple ingredients, which act on multiple targets [4]. Shimotsuto (called *Si*-*Wu*-*Tang* in Chinese medicine) is a traditional Japanese Kampo medicine used to treat gynecological diseases. It comprises four crude drugs: Angelicae Acutilobae Radix, Paeoniae Radix, Rehmanniae Radix, and Cnidii Rhizoma (see Supplementary Table 1 for the original plant source and medicinal part of each crude drug). Shimotsuto is approved by the Japanese Ministry of Health, Labour and Welfare as a prescription drug for relief from irregular menstruation, oversensitivity to cold, and chilblains. Part of the pharmacological actions of shimotsuto is believed to be improvement of blood and the circulatory system. The pharmacological action has been shown by not only historical interpretation but also recent clinical and basic studies. For example, shimotsuto significantly increases the microcirculation of the bulbar conjunctiva [5], and some of its ingredients (e.g., senkyunolide A, butylphthalide, and ligustilide) [6, 7] induce vasodilatation and vasorelaxation [8, 9], while others (e.g., paeoniflorin) have antiplatelet and anticoagulation effects [10]. Although the ingredients may be the active ingredients in shimotsuto, it is unclear whether they are absorbed into the body after oral administration of shimotsuto. Therefore, they are only considered as candidates. The pharmacokinetic information is essential for identifying which ingredients of shimotsuto are active.

This study investigated the pharmacokinetics of shimotsuto’s ingredients following oral administration. We administered shimotsuto orally to rats and then measured the plasma levels of seven candidate active ingredients using targeted liquid chromatography–tandem mass spectrometry (LC–MS/MS). Second, we performed nontargeted liquid chromatography–high-resolution mass spectrometry (LC–HRMS) analysis of rat plasma following administration of constituent crude drugs of shimotsuto to extensively explore blood-absorbed ingredients of shimotsuto. Finally, we quantified the plasma levels of two compounds detected and identified by nontargeted LC–HRMS analysis.

## Materials and methods

### Test substances and reagents

The extract powders of shimotsuto (lot no. 361142800) and its constituent crude drugs (Angelicae Acutilobae Radix [lot no. 2131002010], Paeoniae Radix [lot no. 2131001010], Rehmanniae Radix [lot no. 2151011010], and Cnidii Rhizoma [lot no. 2131004010]) were supplied by Tsumura & Co. (Tokyo, Japan). These extract powders were produced by spray-drying of a hot water extract mixture of the constituent crude drugs, and their qualities were standardized and guaranteed by measuring several characteristic marker ingredients on the basis of good manufacturing practices, as defined by the Japanese Ministry of Health, Labour and Welfare. Supplementary Figure 1 shows the 3D HPLC profile of each extract powder provided by Tsumura & Co.

The standard substances of ingredients contained in shimotsuto were obtained as follows: catalpol, paeoniflorin, albiflorin, ferulic acid, ligustilide, senkyunolide A, and butylphthalide from Tsumura & Co. and bergapten from Chem Faces (Hubei, China), 8-debenzolypaeoniflorin from BioBioPha (Yunnan, China). In addition, niflumic acid, imperatorin, and swertiamarin were obtained from Sigma-Aldrich (St. Louis, MO, USA), Tsumura & Co., and Fujifilm Wako Pure Chemical Industries (Osaka, Japan), respectively, as internal standards for quantitative (targeted) analysis. All other chemicals were purchased from commercial sources.

### Animals

Six-week-old male Sprague–Dawley rats were purchased from Charles River Laboratories (Yokohama, Japan). The rats were housed in cages at 23 °C ± 3 °C with a relative humidity of 50% ± 20% in a 12/12 h light/dark cycle. They were allowed free access to water and standard laboratory food (MF, Oriental Yeast Co., Ltd., Tokyo, Japan). The rats were used in experiments following habituation for several days.

### Ethics

Planning was according to the guidelines for animal care and use of laboratory animals. The study was approved by the Experimental Animal Ethics Committees of Tsumura & Co. (approval no. and date, 17-084/February 21, 2018).

### Quantitative (targeted) analysis of the ingredients in plasma after shimotsuto administration

#### Shimotsuto administration and plasma sample collection

The rats were fasted for 16 h and then orally administered shimotsuto extract powder suspended in distilled water at a dosage of 1 g/10 mL/kg. The animals (*n* = 3/time point) were sacrificed at 0.25, 0.5, 1, 2, 4, 6, 10, or 24 h under anesthesia with isoflurane after drug administration by collecting whole blood from the abdominal inferior vena cava using heparinized syringes. The blood samples were centrifuged at 1700×*g* for 15 min at 4 °C to obtain plasma, and the plasma samples were stored at less than − 75 °C until LC–MS/MS analysis.

Blank plasma samples were also obtained from normal rats without drug administration after fasting for approximately 16 h using the same procedure. These samples were used to generate calibration curves for quantifying plasma levels of shimotsuto ingredients in LC–MS/MS analysis.

#### LC–MS/MS analysis of plasma samples

The plasma levels of nine ingredients were quantified using quantitative (targeted) analysis. Among them, seven ingredients (catalpol, paeoniflorin, albiflorin, senkyunolide A, butylphthalide, ferulic acid, and ligustilide) are candidate active ingredients in shimotsuto (Table 1). Two ingredients (bergapten and 8-debenzoylpaeoniflorin) are shimotsuto ingredients which were detected and identified in shimotsuto-treated rat plasma using nontargeted LC–HRMS analysis. The nine ingredients were quantified by collecting plasma samples at 0–24 h after shimotsuto administration, pretreating with the following procedures, and then analyzing by targeted LC–MS/MS using selected reaction-monitoring mode. The LC–MS/MS system was composed of a triple quadruple MS (TripleQuad6500; SCIEX, Framingham, MA, USA) and an Agilent 1290 Infinity LC system (Agilent Technologies, Santa Clara, CA, USA). For highly sensitive and selective quantitative (targeted) analysis of each compound, we preliminarily examined optimal pretreatment and analytical LC–MS/MS methods (e.g., MS parameters and LC conditions).

**Table 1:** Pharmacological action of the ingredients contained in shimotsuto related to improvement of blood and the circulatory system

Crude drug	Compound	Pharmacological action related to improvement of blood and the circulatory system
Rehmanniae radix	Catalpol	Protective effect on vascular endothelial function via multiple actions such as induction of serum nitric oxide [11]
Paeoniae radix	Paeoniflorin	Reduction of blood viscosity via antithrombotic effect in rat model [12]
	Albiflorin	Reduction of blood viscosity via antithrombotic effect in rat model [12]
Angelicae Acutilobae Radix and Cnidii Rhizome	Senkyunolide A	Vasorelaxation [8], vasorelaxation and increased blood flow effects [13]
	Butylphthalide	Vasodilation [9], vasorelaxation and increased blood flow effects [13]
	Ferulic acid	Vasodilation effect [14]
	Ligustilide	Activation of transient receptor potential ankyrin 1 (TRPA1; which plays a role in vasodilation) [15], vasorelaxation [8], vasorelaxation and increased blood flow effects [13]

For quantification of catalpol, paeoniflorin, albiflorin, ferulic acid, and senkyunolide A in plasma, 100 µL of plasma sample was mixed with 20 µL of methanol (or working solution) and 20 µL of internal standard (niflumic acid or swertiamarin) and pretreated by deproteinization using 500 µL of acetonitrile. After centrifugation at 7000×*g* for 5 min at 4 °C, the whole volume of supernatant was collected and then dried under a stream of nitrogen gas. Finally, the dried residue was dissolved in 60 µL of the specific HPLC mobile phase used for analysis and then a 10 μL aliquot was injected into the LC–MS/MS system.

For quantification of ligustilide and butylphthalide in plasma, 200 µL of plasma sample was mixed with 40 µL of methanol (or working solution) and 40 µL of internal standard (imperatorin) and centrifuged at 15000×*g* for 10 min at 4 °C, the whole volume of supernatant was collected and subsequently pretreated by solid-phase extraction using Oasis HLB Extraction Cartridge (Waters, Milford, MA, USA). The acetonitrile elutes were collected and then dried under a steam of nitrogen gas. Finally, the dried residue was dissolved in 70 µL of the specific HPLC mobile phase and injected 20 µL into the LC–MS/MS system.

For quantification of bergapten in plasma, 100 µL of plasma sample was mixed with 20 µL of methanol (or working solution) and 20 µL of internal standard (niflumic acid) and pretreated by solid-phase extraction using an Oasis HLB 96-well µElution plate. Next, the mixied elutes of acetonitrile and isopropyl alcohol (1:1, v/v) were collected and then dried under a steam of nitrogen gas. Finally, the dried residue was dissolved in 60 µL of the specific HPLC mobile phase and injected 10 µL into the LC–MS/MS system.

For quantification of 8-debenzoylpaeoniflorin in plasma, 100 µL of plasma sample was mixed with 20 µL of methanol (or working solution) and 20 µL of internal standard (niflumic acid) and pretreated by liquid–liquid extraction using 500 µL of ethyl acetate. After centrifugation at 7000×*g* for 5 min at 4 °C, the whole volume of supernatant was collected and then dried under a stream of nitrogen. The dried residue was dissolved in 60 µL of the specific HPLC mobile phase and injected 10 µL into the LC–MS/MS system.

Supplementary Table 2 shows an original database including 136 ingredients of shimotsuto used in nontargeted LC–HRMS analysis. Supplementary Tables 3–6 specify the analytical conditions of LC–MS/MS and the range of quantification and correlation coefficient of each calibration curves.

In addition, pharmacokinetic parameters of the shimotsuto ingredients detected in the plasma, such as the maximum concentration (*C*_max_) and area under the plasma level–time curve (AUC_*0−last*_), were calculated by noncompartmental moment analysis using Phoenix WinNonlin software (Certara L.P., St. Louis, MO, USA).

### Qualitative (nontargeted) analysis of shimotsuto ingredients in plasma

#### Constituent crude drug of shimotsuto administration and plasma sample collection

The rats were fasted for 16 h and then orally administered Angelicae Acutilobae Radix, Paeoniae Radix, Rehmanniae Radix, or Cnidii Rhizoma extract powder suspended in distilled water at a dosage of 1 g/10 mL/kg. After the administration, the animals (*n* = 2/time point) were sacrificed at 1 or 10 h under anesthesia with isoflurane after drug administration by collecting whole blood from the abdominal inferior vena cava using heparinized syringes. The two sampling points (1 and 10 h) were set to extensively explore both fast- and slow-absorbing ingredients. The blood samples were centrifuged at 1700×*g* for 15 min at 4 °C to obtain plasma, and the plasma samples were stored at less than − 75 °C until LC–HRMS analysis.

#### LC–HRMS analysis of plasma samples

Before LC–HRMS analysis, the plasma samples were deproteinized using methanol as follows: 100 µL of plasma sample was mixed with 700 µL of methanol and then vortexed for 2 min. After centrifugation at 7000×*g* for 5 min at 4 °C, the supernatant was collected and then dried using a centrifugal evaporator, dissolved the dried residue in 80 μL of the initial mobile phase of LC, and recentrifuged at 7000×*g* for 5 min at 4 °C. Finally, a 5 μL aliquot of the supernatant was injected into the LC–HRMS system and analyzed in both positive and negative ion modes.

We interfaced the Acquity Ultra-Performance Liquid Chromatography System (Waters) to a Xevo G2-XS quadrupole time-of-flight MS system (Waters) equipped with an electrospray ionization probe in sensitivity mode. All ions within *m*/*z* = 50–1000 were acquired by the MS^E^ method (data-independent acquisition). The MS^E^ method enables us to simultaneously acquire information from both precursor and product ions; it is widely used for analyzing multiple compounds in complex samples [16, 17]. We created two acquisition functions with different collision energies: a low-energy function with 6 eV collision energy and a high-energy function with 15–40 eV collision energy. The mass spectrometer resolution was set at more than 20,000. Supplementary Table 7 details the HPLC conditions of LC–HRMS.

## Results and discussion

### Quantitative (targeted) analysis of seven candidate active ingredients of shimotsuto in shimotsuto-treated rat plasma

Several in vivo and in vitro assays have reported that shimotsuto’s multiple ingredients have pharmacological actions related to improvement of blood and the circulatory system (Table 1). However, because of a lack of pharmacokinetic information about shimotsuto, it is unclear whether these ingredients are active in in vivo conditions.

**Fig. 1:**
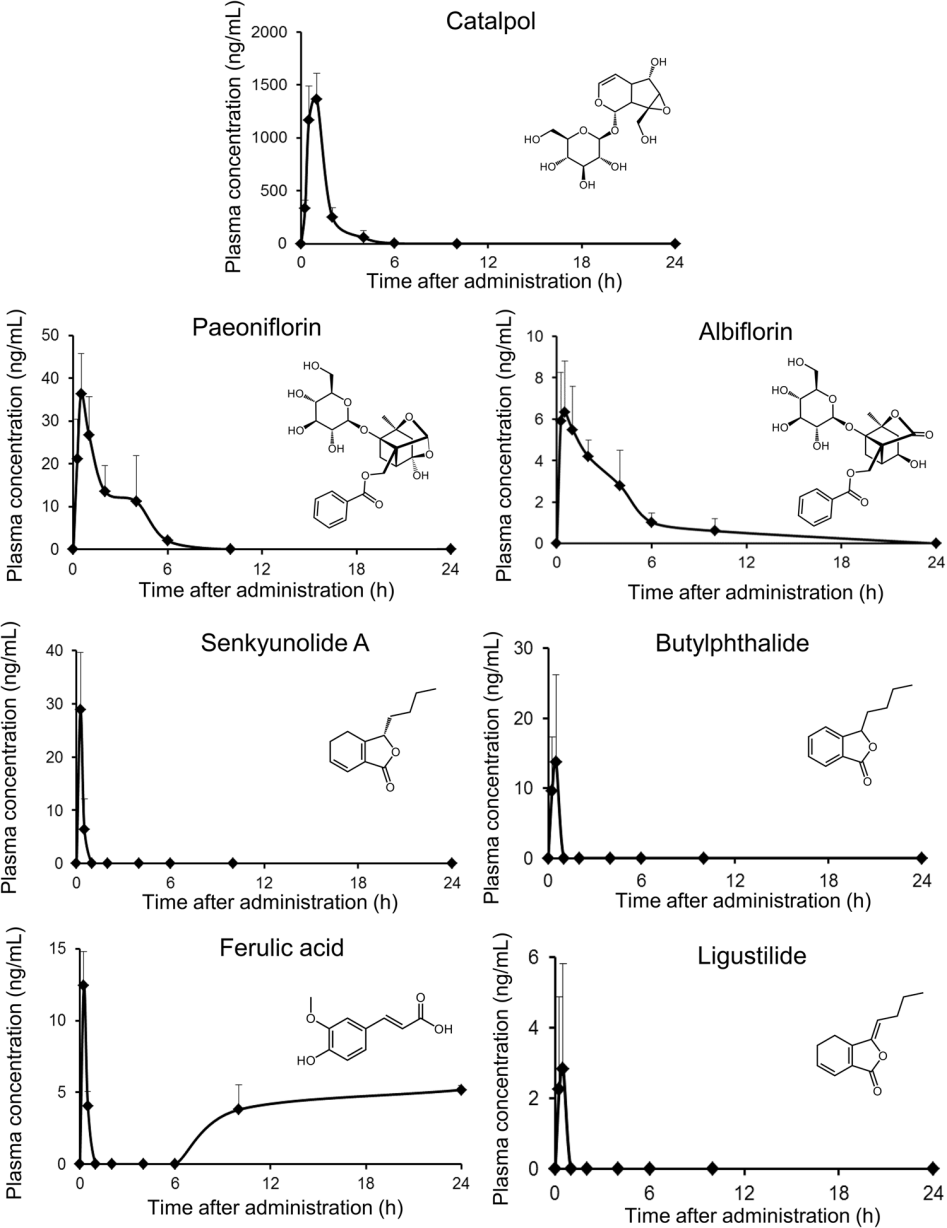
Plasma level–time curves of seven shimotsuto ingredients. Each plasma sample was obtained from whole rat blood after oral administration of shimotsuto at a dosage of 1 g/kg and was analyzed by LC–MS/MS using the selected reaction-monitoring mode. Individual points represent the mean + SD for three independent animals

We detected all seven candidate active ingredients (catalpol, paeoniflorin, albiflorin, ligustilide, senkyunolide A, butylphthalide, and ferulic acid) in plasma following oral administration of shimotsuto. Figure 1 and Table 2 show the temporal plasma levels and pharmacokinetic parameters, respectively. Catalpol had the highest *C*_max_ (1360 ng/mL), followed by paeoniflorin (36.3 ng/mL) and senkyunolide A (28.9 ng/mL). The pharmacology, pharmacokinetics, and safety concerns of catalpol, an iridoid glucoside, have been widely studied [18]. The absolute bioavailability of catalpol is 66.7% [18], indicating that it is well absorbed from the gut lumen and is transferred to the circulating blood with little or no intestinal and hepatic metabolism, although it is water soluble (log *P* = −3.15 by ChemDraw software). However, in our unpublished data, the plasma levels of catalpol in humans after goshajinkigan, including Rehmanniae Radix, administration were quite low, although the plasma levels in rats were high, the same as plasma levels in rats following shimotsuto administration. The factor of species difference in the pharmacokinetics is unfortunately unclear; however, we consider that the contributions of intestinal and/or hepatic metabolism in humans are higher compared to rats.

**Table 2:** Pharmacokinetic parameters of seven shimotsuto ingredients

Compound	*C*_max_ (ng/mL)	*t*_max_ (h)	AUC_*0−last*_ (ng h/mL)	*t*_1/2_ (h)
Catalpol	1360	1.0	2030	0.643
Paeoniflorin	36.3	0.50	83.6	1.48
Senkyunolide A	28.9	0.25	8.04	–
Butylphthalide	13.7	0.50	4.12	–
Ferulic acid	12.4	0.25	75.0	–
Albiflorin	6.34	0.50	24.2	2.71
Ligustilide	2.83	0.50	0.916	–

–, Not calculated because of a deficit of measured points during the elimination phase

*C*_max_, maximum concentration; *t*_max_, time to maximum concentration; AUC_*0−last*_, area under the plasma level–time curve from 0 to final observation time; *t*_1/2_, apparent elimination half-life

All values were calculated by the mean concentration (*n* = 3) per time point using Phoenix WinNonlin software (Certara L.P.)

Paeoniflorin is one of marker ingredients used for quality confirmation of shimotsuto, and albiflorin is its isomer. Although the paeoniflorin content in shimotsuto is high [7], in this study, paeoniflorin had a much lower *C*_max_ compared to catalpol but a higher *C*_max_ compared to albiflorin (6.34 ng/mL) (Fig. 1 and Table 2). This result was consistent with the reported low bioavailability of paeoniflorin in rats [19]. Similar plasma level–time curves of both paeoniflorin and albiflorin indicated that the two compounds are quickly absorbed and quickly eliminated. In the elimination phase of paeoniflorin, the lagged point was at 4-h post-administration, consistent with previous reports [19, 20]. This result is probably due to enterohepatic circulation, because the cumulative biliary excretion after oral administration of paeoniflorin is 1.3% of the dose within 24 h [19]. In addition, multiple absorption sites or ingredient–ingredient interaction might affect plasma paeoniflorin levels in the elimination phase.

The plasma levels of senkyunolide A, butylphthalide, and ligustilide (phthalides from Angelicae Acutilobae Radix and Cnidii Rhizome) were lower compared to catalpol and paeoniflorin (Fig. 1 and Table 2). The oral bioavailability of ligustilide is low (2.6%) [21], which is consistent with our results. The low bioavailability indicates a low absorption rate in the intestine and a high metabolism rate in the intestine and liver. Phthalides are believed to be metabolized into butylidenephthalide via intermediary metabolites, such as senkyunolide I and senkyunolide J [22]. Therefore, after being metabolized in the liver, phthalides might be mainly present in plasma as metabolites.

The pharmacokinetic profile of ferulic acid was unique compared to other ingredients analyzed. Ferulic acid was quickly absorbed then also quickly and completely eliminated, and it subsequently reappeared in the plasma 10- and 24-h post-administration. Although the reason was not investigated, the profile may indicate several pharmacokinetic characteristics (e.g., enterohepatic circulation) of ferulic acid. The occurrence of a peak in the elimination phase is often observed when a compound is reabsorbed by enterohepatic circulation. However, such a peak was not observed following sole administration of ferulic acid [23], indicating that enterohepatic circulation might not be responsible for the pharmacokinetic profile of ferulic acid following shimotsuto administration. Chlorogenic acid contained in shimotsuto’s constituent crude drugs (Angelica Acutilobae Radix and Cnidii Rhizoma) [24] has been reported to be metabolized into ferulic acid in the living body [25]. Therefore, it may be the reason for ferulic acid appearing in the late phase as a metabolite from coexistent ingredients of shimotsuto. However, further studies are required to understand this phenomenon.

### Qualitative (nontargeted) analysis of plasma compounds

**Fig. 2:**
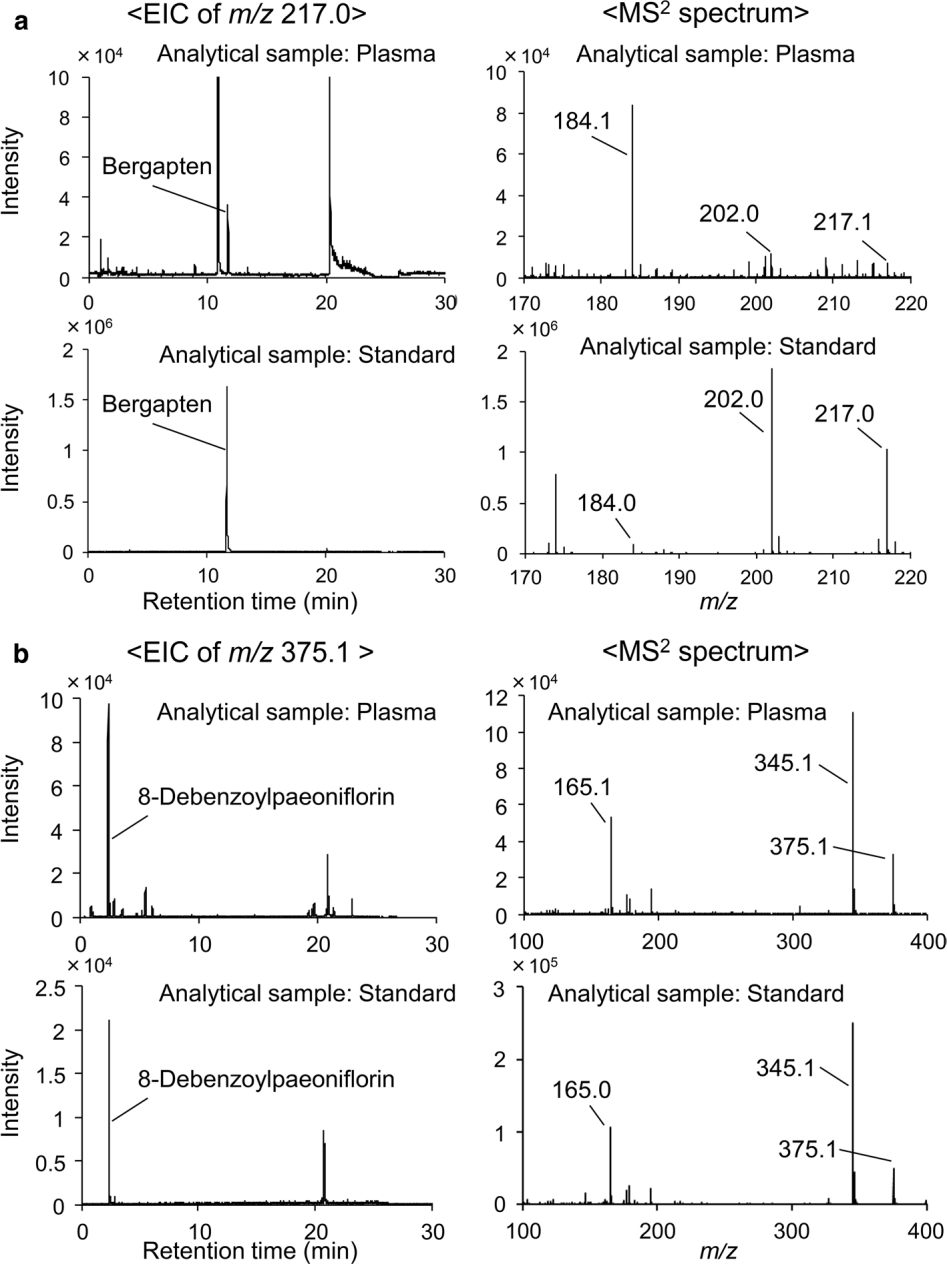
Identification results of plasma bergapten (**a**) and 8-debenzolylpaeoniflorin (**b**) by comparison of extracted ion chromatogram and MS^2^ spectrum between the plasma sample and the standard sample. The plasma sample of rats at 1 h after administration of Angelicae Acutilobae Radix or Paeoniae Radix was used for LC–HRMS identification of each bergapten and 8-debenzolylpaeoniflorin

In targeted analysis, the seven candidate active ingredients were selected and analyzed; however, more ingredients might be absorbed following administration and can participate in the pharmacological action of shimotsuto. Nontargeted LC–HRMS allows wide-ranging exploration of compounds absorbed in the plasma. Recently, we conducted a pharmacokinetic study of the Kampo medicine maobushisaishinto using nontargeted analysis with an original database containing chemical information (e.g., monoisotopic mass and structural formula) of ingredients and identified a few bioavailable compounds [26]. In this study, nontargeted LC–HRMS analysis assigned seven shimotsuto ingredients except for ingredients analyzed by targeted analysis to detected peaks (Table 3). The seven ingredients were estimated by comparing their observed masses and characteristic fragment ions with theoretical values, and the standard substances of six ingredients (not paeonilactone C) were prepared and analyzed by the same method as the plasma analysis. The retention times and characteristic fragment ions of the peaks detected from the standard substances of 8-debenzoylpaeoniflorin and bergapten corresponded to those of the peaks detected from plasma samples (Fig. 2). 8-Debenzoylpaeoniflorin is known as an ingredient of Paeoniae Radix, but a minor compound among ingredients contained in Paeoniae Radix. Therefore, plasma 8-debenzoylpaeoniflorin is considered to exist as a metabolite. A recent pharmacokinetic study on rats had reported the presence of not only 8-debenzoylpaeoniflorin but also 8-debenzoylalbiflorin and demonstrated that the former is produced from both paeoniflorin and albiflorin, whereas the latter is produced only from albiflorin [27]. The fact that we detected 8-debenzoylpaeoniflorin but not 8-debenzoylalbiflorin (Fig. 2b) in shimotsuto-treated rat plasma may be attributed to the abundance of precursors (paeoniflorin and albiflorin) present in shimotsuto. Bergapten has been detected in the plasma of rat orally administered the extract of Angelicae dahuricae Radix, which is also found in *Apiaceae* plants [28]. This result is consistent with our detection of bergapten in Angelicae Acutilobae Radix-treated rat plasma by nontargeted LC–HRMS analysis. The peaks of catechin, 3-butylidenephthalide, and eugenin were detected by analysis of standard substances, but the retention time did not correspond to the peaks detected in the plasma. In contrast, no peaks were detected in the analysis of standard coniferyl ferulate. The peaks attributed to catechin, 3-butylidenephthalide, eugenin, and coniferyl ferulate in the plasma samples are currently unidentified, and further studies are required to identify them.

**Table 3:** Ingredients detected in rat plasma after administration of each constituent crude drug of shimotsuto using nontargeted LC–HRMS

*t*_R_ (min)	Adduct	Observed mass (Da)	Error (ppm)	Molecular formula	Assigned name from our chemical database	Plasma collected 1 h postadministration^a^	Plasma collected 10 h postadministration^a^	Detected fragment ions
*Administration of Paeoniae Radix*								
2.39	− H	376.1357	− 3.4	C_16_H_24_O_10_	8-Debenzoylpaeoniflorin	++	+	99.9250, 160.8403, 162.8372, 165.0543, 177.0555, 195.0635, 345.1172, 346.1199
4.19	+ H	290.0779	− 3.9	C_15_H_14_O_6_	Catechin	++	Not detected	116.9750, 123.0427, 136.0602, 137.0586, 139.0378, 139.9615, 147.0421, 165.0526, 183.9309, 273.0737, 282.2193
5.84	+ H	318.1103	− 0.2	C_17_H_18_O_6_	Paeonilactone C	+	Not detected	105.0332, 133.0641, 151.0749, 161.0587, 179.0695, 197.0806, 198.0836, 301.1067
*Administration of Angelicae Acutilobae Radix*								
11.73	+ H	216.0416	− 3.3	C_12_H_8_O_4_	Bergapten	+	Not detected	126.0212, 174.0295, 202.0249, 208.0624, 215.1047
*Administration of Cnidii Rhizoma*								
10.55	− H	356.1249	− 3.2	C_20_H_20_O_6_	Coniferyl ferulate	++	+	146.9651, 281.0802, 296.1037, 311.1278
10.55	+ H	206.0569	− 4.6	C_11_H_10_O_4_	Eugenin	++	Not detected	103.0529, 119.0476, 131.0478, 137.0585, 147.0428, 161.0574, 163.0737, 175.0374, 176.9967, 189.0524, 204.9736
11.66	+ H	188.0828	− 5.1	C_12_H_12_O_2_	3-Butylidenephthalide	++	Not detected	159.1156

*t*_R_, Retention time

^a^The number of + signs represent the sample number in which the ingredient was detected in the plasma after administration of each constituent crude drug of shimotsuto (*n *= 2 per sampling time per crude drug). Plasma samples were obtained from whole rat blood 1 or 10 h after oral administration of each constituent crude drug of shimotsuto at a dosage of 1 g/kg

### Quantitative (targeted) analysis of bergapten and 8-debenzoylpaeoniflorin in shimotsuto-treated rat plasma

**Fig. 3:**
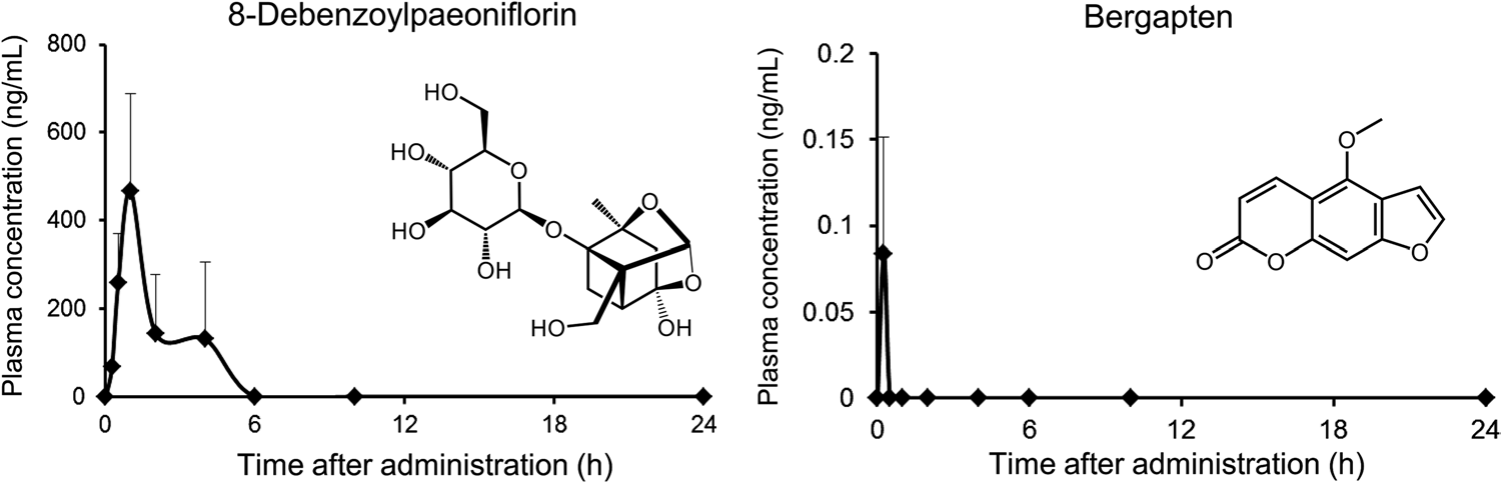
Plasma level–time curves of bergapten and 8-debenzolylpaeoniflorin. Each plasma sample was obtained from whole rat blood after oral administration of shimotsuto at a dosage of 1 g/kg and was analyzed by LC–MS/MS using the selected reaction-monitoring mode. Individual points represent the mean + SD for three independent animals

To quantify the plasma concentrations of two compounds (bergapten and 8-debenzoylpaeoniflorin) identified in nontargeted analysis, quantitative (targeted) analysis was performed. The plasma concentration of bergapten was low, with a *C*_max_ of 0.0837 ng/mL (Fig. 3 and Table 4). In contrast, the *C*_max_ of 8-debenzoylpaeoniflorin was comparatively high (466 ng/mL) compared to other shimotsuto ingredients. As described earlier, 8-debenzoylpaeoniflorin has been reported to be a metabolite of paeoniflorin and albiflorin [27]; however, there are no reports on the presence of 8-debenzoylpaeoniflorin in the blood following administration of Paeoniae Radix. The *C*_max_ of 8-debenzoylpaeoniflorin in plasma following shimotsuto administration was approximately tenfold compared to paeoniflorin, which is a major compound in Paeoniae Radix and is well measured in pharmacokinetic studies of herbal medicines containing it. To the best of our knowledge, this is first report to show plasma levels of 8-debenzoylpaeoniflorin after administration of a herbal medicine containing Paeoniae Radix. The plasma concentration–time profile of 8-debenzoylpaeoniflorin was bimodal (Fig. 3), indicating that this compound, like paeoniflorin, may also be affected by enterohepatic circulation. However, because Paeoniae Radix also contains 8-debenzoylpaeoniflorin [29], the first peak of the bimodal profile may be due to the absorption of 8-debenzoylpaeoniflorin in shimotsuto, whereas the second peak may be a metabolite of paeoniflorin and albiflorin. Further studies are required to reveal the pharmacokinetics of 8-debenzoylpaeoniflorin in detail.

**Table 4:** Pharmacokinetic parameters of bergapten and 8-debenzoylpaeoniflorin

Ingredient	*C*_max_ (ng/mL)	*t*_max_ (h)	AUC_*0−last*_ (ng h/mL)	*t*_1/2_ (h)
8-Debenzoylpaeoniflorin	466	1.0	811	-
Bergapten	0.0837	0.25	0.0105	–

–, Not calculated because of a deficit of measured points during the elimination phase

*C*_max_, maximum concentration; *t*_max_, time to maximum concentration; AUC_*0−last*_, area under the plasma level–time curve from 0 to final observation time; *t*_1/2_, apparent elimination half-life

All values were calculated by the mean concentration (*n* = 3) per time point using Phoenix WinNonlin software (Certara L.P.)

### Consideration of pharmacological actions of bioavailable shimotsuto ingredients against gynecological diseases

As mentioned earlier, part of the pharmacological action of shimotsuto is improvement of blood and the circulatory system. The shimotsuto ingredients quantified in this study have multiple pharmacological actions. For example, catalpol administered orally inhibits apotosis of the ovarian granulocytes of aged female rats, indicating that catalpol works on sex organs by nourishing ovarian tissue and improving the quality and quantity of ovarian follicles [30]. Catalpol also has an anti-inflammatory effect on lipopolysaccharide-induced endometritis by inhibiting inflammation and toll-like receptor 4/nuclear factor kappa B signaling [31]. Paeoniflorin ameliorates ovarian functions by inhibiting testosterone production in rat ovaries [32]. The pharmacological action of 8-debenzoylpaeoniflorin, whose *C*_max_ was the second highest in this study, is not well studied. We recently performed a screening assay against transient receptor potential (TRP) channels for in vitro pharmacological profiling of phytochemicals [26] with 8-debenzoylpaeoniflorin and other bioavailable shimotsuto ingredients using TRP stable expression cell lines. 8-Debenzoylpaeoniflorin slightly activated transient receptor potential ankyrin 1 (TRPA1) (the test methods and results are described in Supplementary file 1 and supplementary Table 8, respectively). Future studies are required to determine more detail pharmacological actions of 8-debenzoylpaeoniflorin. Senkyunolide A, ligustilide, and buthylphthalide potently activated TRPA1 (supplementary Table 8), although their half-maximal effective concentration (EC_50_) was higher than their *C*_max_ after oral administration of shimotsuto.

Overall, our results showed that the pharmacological action of shimotsuto is exerted by its multiple bioavailable ingredients.

## Conclusion

The seven candidate active ingredients (catalpol, paeoniflorin, albiflorin, ligustilide, senkyunolide A, butylphthalide, and ferulic acid) related to improvement of blood and the circulatory system by shimotsuto were detected in plasma after oral administration of shimotsuto, and catalpol has the highest *C*_max_, followed by paeoniflorin. Furthermore, two bioavailable shimotsuto ingredients (bergapten and 8-debenzoylpaeoniflorin) were detected (using nontargeted analysis) in rat plasma, which were subsequently quantified. Of the total nine ingredients quantified plasma concentration, 8-debenzoylpaeoniflorin showed the second highest *C*_max_ after catalpol. The results indicate that the nine ingredients are absorbed into the blood after oral administration of shimotsuto and possibly contribute to its pharmacological action, and are also presumably useful for understanding the scientific evidence of shimotsuto as a remedy against gynecological diseases.

## Supplementary Information

Below is the link to the electronic supplementary material.

**Supplementary Fig.** **1** 3D HPLC profile of shimotsuto (a), Rehmanniae Radix (b), Paeoniae Radix (c), Cnidii Rhizome (d), and Angelicae Acutilobae Radix (e) extract powders. Each peak in the HPLC profile was identified by comparison of the retention times and UV spectra of chemically defined standard compounds. (JPEG 871 kb)

Supplementary material 2 (JPEG 873 kb)

Supplementary material 3 (JPEG 801 kb)

Supplementary material 4 (JPEG 699 kb)

Supplementary material 5 (JPEG 766 kb)

**Supplementary File 1** Experimental methods for evaluation of agonistic action of shimotsuto ingredients against TRP channels (DOCX 32 kb)

**Supplementary Table** **1** Original plant source, medicinal part, and composition ratio of each constituent crude drug of shimotsuto, **Supplementary Table** **2** Shimotsuto ingredients incorporated into UNIFI software used in nontargeted LC–HRMS, **Supplementary Table** **3** Targeted LC–MS/MS methods: ion parameters of test ingredients of shimotsuto, **Supplementary Table** **4** Targeted LC–MS/MS methods: ion source parameters in LC–MS/MS analysis, **Supplementary Table** **5** Targeted LC–MS/MS methods: HPLC conditions, **Supplementary Table** **6** Calibration curves used for quantification of shimotsuto ingredients, **Supplementary Table** **7** HPLC conditions of nontargeted LC–HRMS, **Supplementary Table** **8** Agonistic effect of shimotsuto ingredients against TRP channels (DOCX 51 kb)
