# Supplementary figures and images for: Pharmacokinetic study of traditional Japanese Kampo medicine shimotsuto used to treat gynecological diseases in rats
Source: J Nat Med. 2021 Jan 4;75(2):361–71. doi: 10.1007/s11418-020-01474-x (PMC7902330; doi:10.1007/s11418-020-01474-x)

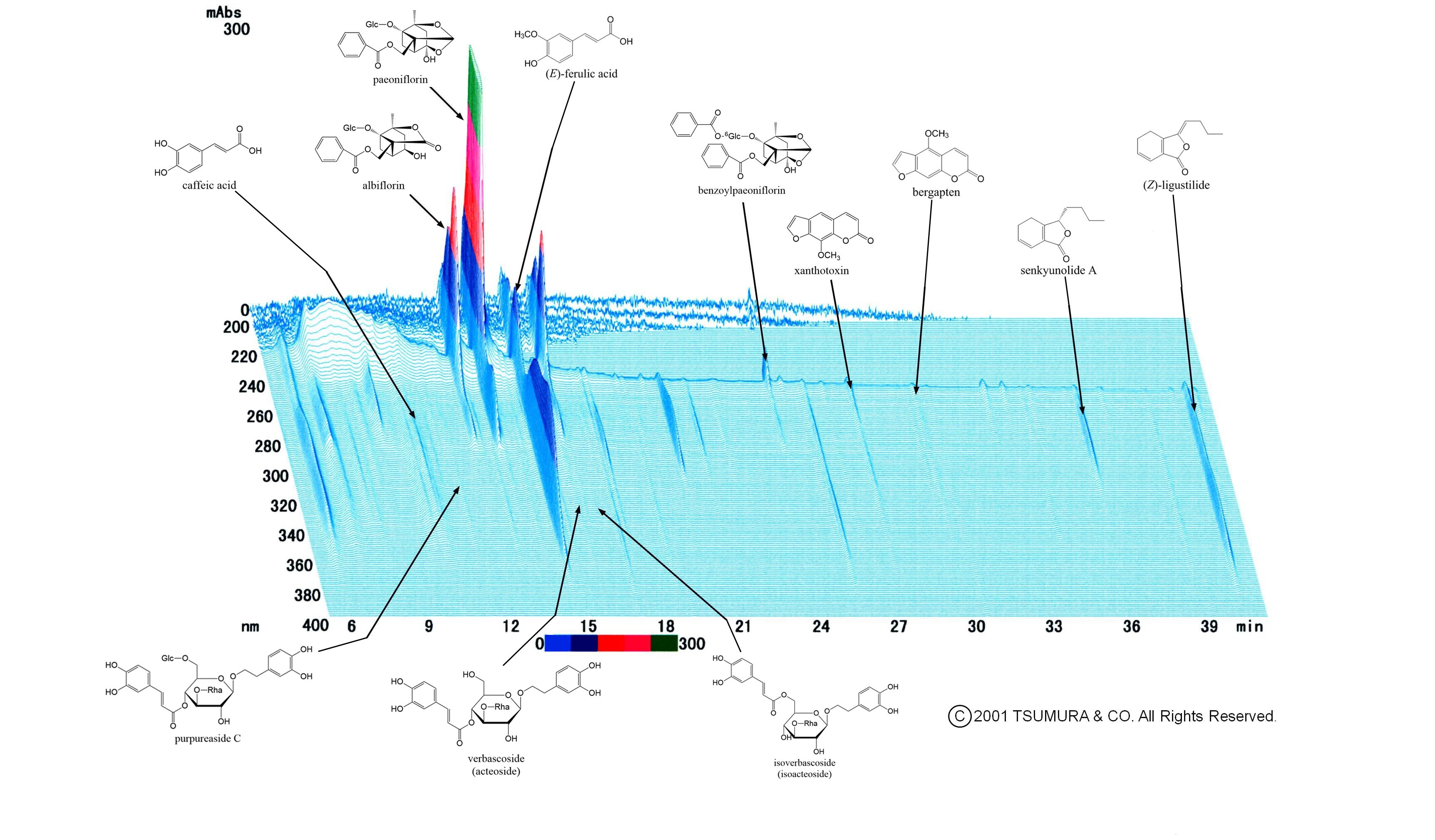

Supplement: Supplementary file 1 — Supplementary Fig. 1 3D HPLC profile of shimotsuto (a), Rehmanniae Radix (b), Paeoniae Radix (c), Cnidii Rhizome (d), and Angelicae Acutilobae Radix (e) extract powders. Each peak in the HPLC profile was identified by comparison of the retention times and UV spectra of chemically defined standard compounds. (JPEG 871 kb) [file 11418_2020_1474_MOESM1_ESM.jpg]

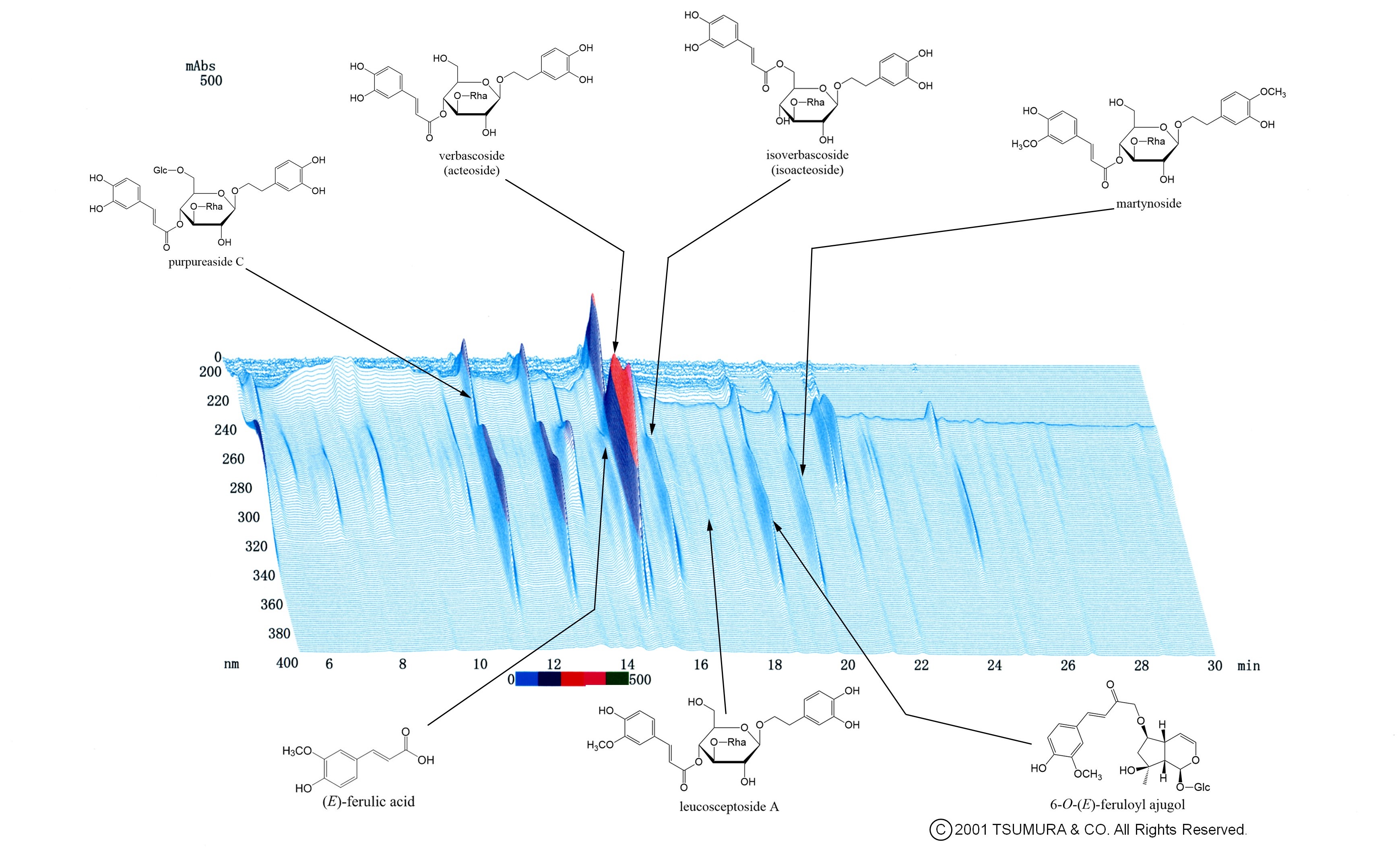

Supplement: Supplementary file 2 — Supplementary material 2 (JPEG 873 kb) [file 11418_2020_1474_MOESM2_ESM.jpg]

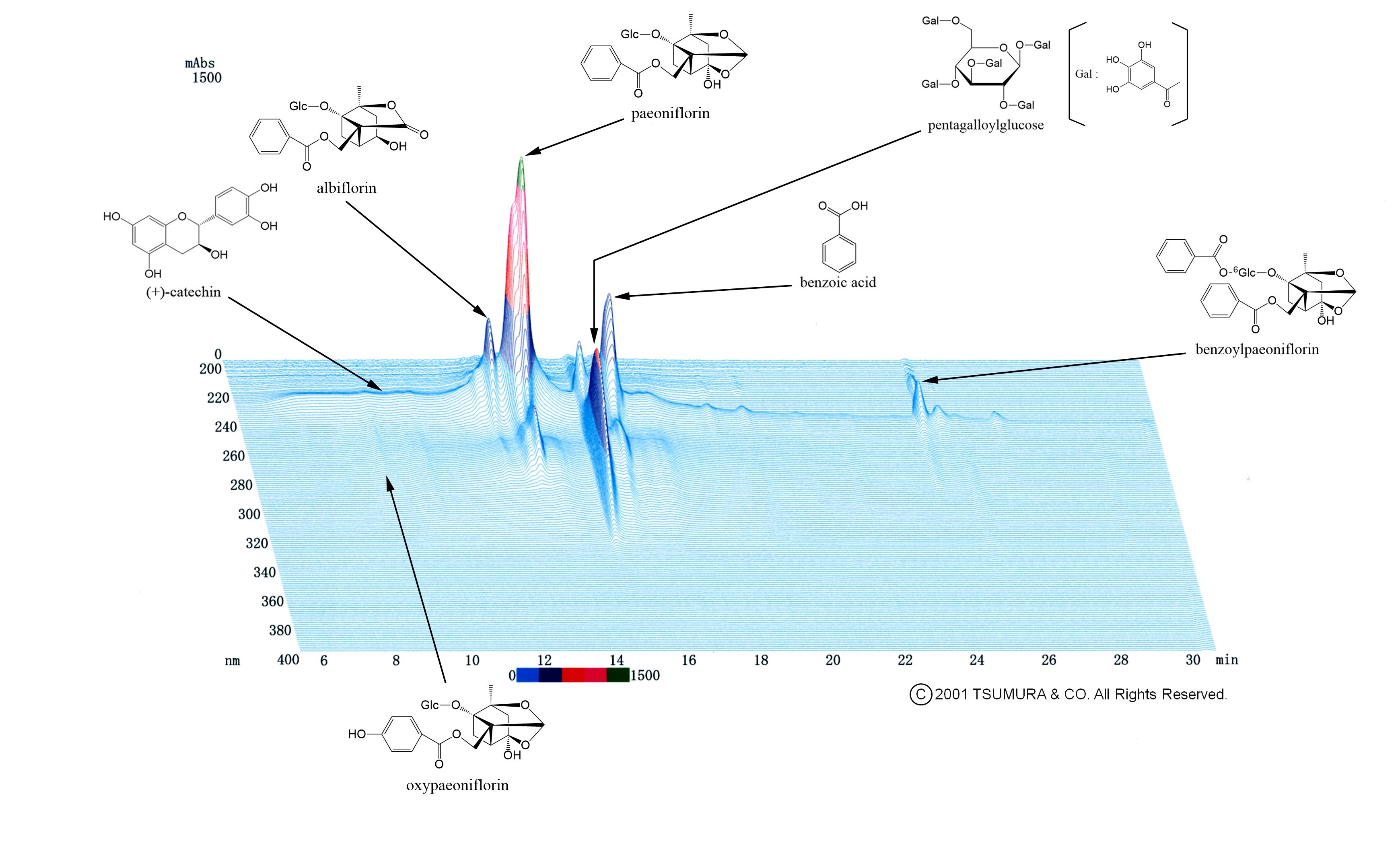

Supplement: Supplementary file 3 — Supplementary material 3 (JPEG 801 kb) [file 11418_2020_1474_MOESM3_ESM.jpg]

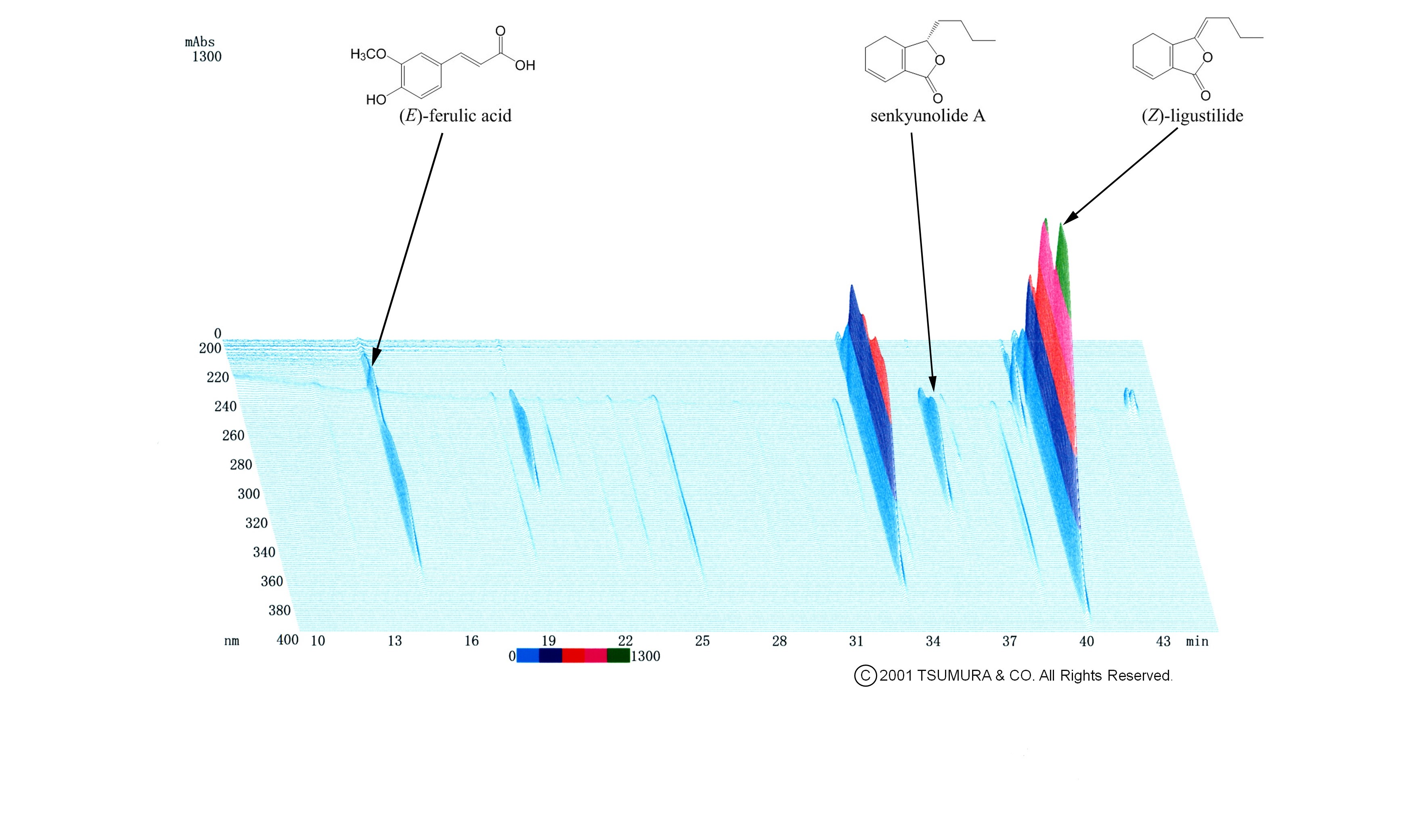

Supplement: Supplementary file 4 — Supplementary material 4 (JPEG 699 kb) [file 11418_2020_1474_MOESM4_ESM.jpg]

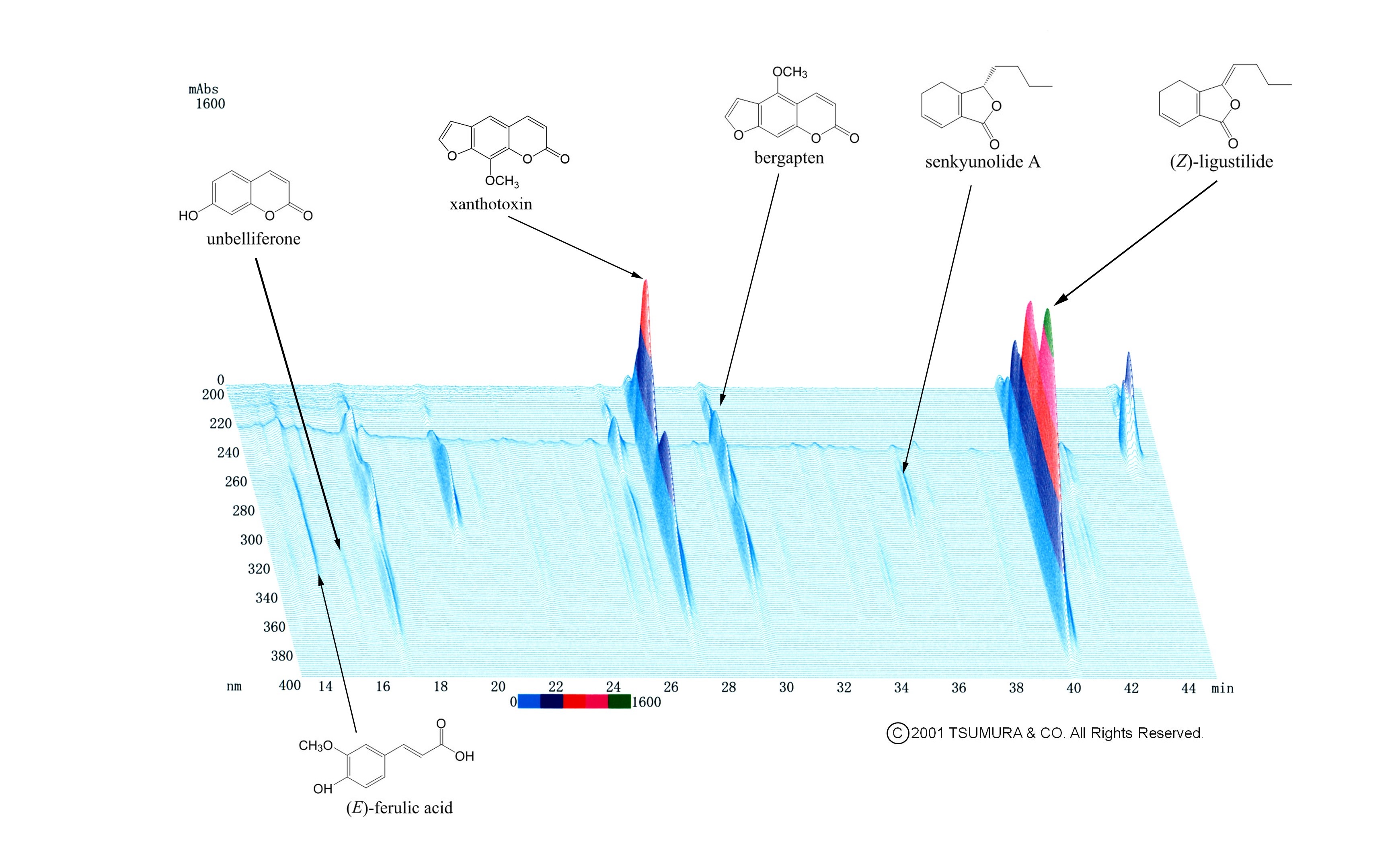

Supplement: Supplementary file 5 — Supplementary material 5 (JPEG 766 kb) [file 11418_2020_1474_MOESM5_ESM.jpg]
