# Supplementary material for: Pharmacokinetic study of traditional Japanese Kampo medicine shimotsuto used to treat gynecological diseases in rats
Source: J Nat Med. 2021 Jan 4;75(2):361–71. doi: 10.1007/s11418-020-01474-x (PMC7902330; doi:10.1007/s11418-020-01474-x)
Supplement: Supplementary file 6 — Supplementary File 1 Experimental methods for evaluation of agonistic action of shimotsuto ingredients against TRP channels (DOCX 32 kb) [file 11418_2020_1474_MOESM6_ESM.docx]

**Supplementary File 1** Experimental methods for evaluation of agonistic action of shimotsuto ingredients against TRP channels

**Methods**

*Plasmid constructs and cell culture*

The full length of each of human transient receptor potential ankyrin 1 (TRPA1), transient receptor potential cation channel subfamily V member 1 (TRPV1), transient receptor potential cation channel subfamily V member 4 (TRPV4), and transient receptor potential cation channel subfamily M member 8 (TRPM8) coding region was introduced into a pcDNA4/TO vector (Thermo Fisher Scientific, Waltham, MA, USA) and transfected into T-REx-293 cells (Thermo Fisher Scientific) to obtain stable cell lines expressing human TRPA1, TRPV1, TRPV4, and TRPM8. Next, the cells were cultured in Dulbecco’s Modified Eagle Medium containing 10% fetal bovine serum (FBS), 4 mM L-glutamine, and penicillin/streptomycin.

*Ca^2+^ influx assay for evaluating TRP channel activity*

First, cells were suspended in culture medium with 1 µg/mL of tetracycline and seeded on a poly-D-lysine-coated 96-well plate at a density of 2.5 × 10^4^ cells/well overnight. Next, the cells were used for Ca^2+^ influx assay, as described previously with minor modifications, using a FLIPR Calcium 5 Assay Kit (Molecular Devices, San Jose, CA, USA) [33]. Briefly, cells were incubated in 80 µL of a Ca^2+^-chelating dye dissolved in assay buffer at pH 7.4 (Hanks’ Balanced Salt Solution containing 20 mmol/L of 4-(2-hydroxyethyl)-1-piperazineethanesulfonic acid [HEPES]) for 30 min at room temperature. Next, the plates were assayed in a FlexStation 3 microplate reader (Molecular Devices). The fluorescence intensity was traced entirely for 80 s, and the fluorescence baseline was measured 20 s before application of 20 µL of the test compound (final concentration = 10 µmol/L). The response was expressed as percentage activation as follows:

Relative fluorescence units (RFU) (% activation) = ([RFU_max_ − RFU_baseline_]/RFU_baseline_) × 100

The activity of each test compound against TRPA1, TRPV1, TRPV4, and TRPM8 was represented as the relative enhancement of the Ca^2+^ influx of each positive control as follows: 20 µmol/L of allyl isothiocyanate (TRPA1 agonist), 2 µmol/L of capsaicin (TRPV1 agonist), 4 µmol/L of GSK1016790A (TRPV4 agonist), and 20 µmol/L of icilin (TRPM8 agonist). The dose–response curve was fitted to the sigmoidal dose–response equation (Top: maximum response of each compound), and EC_50_ was calculated using GraphPad prism 7 software (GraphPad, San Diego, CA, USA).
