# Supplementary material for: Pharmacokinetic study of traditional Japanese Kampo medicine shimotsuto used to treat gynecological diseases in rats
Source: J Nat Med. 2021 Jan 4;75(2):361–71. doi: 10.1007/s11418-020-01474-x (PMC7902330; doi:10.1007/s11418-020-01474-x)
Supplement: Supplementary file 7 — Supplementary Table 1 Original plant source, medicinal part, and composition ratio of each constituent crude drug of shimotsuto, Supplementary Table 2 Shimotsuto ingredients incorporated into UNIFI software used in nontargeted LC–HRMS, Supplementary Table 3 Targeted LC–MS/MS methods: ion parameters of test ingredients of shimotsuto, Supplementary Table 4 Targeted LC–MS/MS methods: ion source parameters in LC–MS/MS analysis, Supplementary Table 5 Targeted LC–MS/MS methods: HPLC conditions, Supplementary Table 6 Calibration curves used for quantification of shimotsuto ingredients, Supplementary Table 7 HPLC conditions of nontargeted LC–HRMS, Supplementary Table 8 Agonistic effect of shimotsuto ingredients against TRP channels (DOCX 51 kb) [file 11418_2020_1474_MOESM7_ESM.docx]

**Supplementary Table 1** Original plant source, medicinal part, and composition ratio of each constituent crude drug of shimotsuto

| **Crude drug** | **Original plant source and medicinal part** | **Composition ratio (%)** |
| --- | --- | --- |
| Angelicae Actilobae Radix | The roots of *Angelica acutiloba* Kitagawa or *Angelica acutiloba* Kitagawa var. *sugiyamae* Hikino | 25 |
| Paeoniae Radix | The roots of *Paeonia lactiflora* Pallas | 25 |
| Rehmanniae Radix | The roots of *Rehmannia glutinosa* Liboschitz var. *purpurea* Makino or *Rehmannia glutinosa* Liboschitz | 25 |
| Cnidii Rhizoma | The Rhizome of *Cnidium officinale* Makino | 25 |

**Supplementary Table 2** Shimotsuto ingredients incorporated into UNIFI software used in nontargeted LC–HRMS

| **Crude drug** | | | |
| --- | --- | --- | --- |
| Rehmanniae Radix | Paeoniae Radix | Cnidii Rhizome | Angelicae Acutilobae Radix |
| Ajugol | Albiflorin | 3-Butyl-3a,4,5,7a-tetrahydro-1(3H)-isobenzofuranone | Angeloylsenkyunolide F |
| Rehmaglutoside A | 6'-Galloylalbiflorin | 3-Butylidene-1(3H)-isobenzofuranone | Acutilobin |
| Rehmaglutoside C | 2'-*O*-Benzoylpaeoniflorin | Senkyunolide J | Falcarindiol |
| Rehmannioside C | 3'-Galloylpaeoniflorin | Senkyunolide A | 7-Hydroxy-6-methoxycoumarin |
| Rehmaglutoside B | 4'-Galloylpaeoniflorin | Senkyunolide G | Tokinolide A |
| 6-O-Vanilloylajugol | 8-Debenzoylpaeoniflorin | Senkyunolide D | Tokinolide B |
| Rehmaglutoside D | p-Digallic acid | Ligustilide | Ferulic acid |
| Rehmannioside B | 11,12-Epoxy-3,23-dihydroxy-30-nor-20(29)-oleanen-28,13-olide | Senkyunolide C | Isosafrole |
| Rehmannioside A | Gnetin H | Senkyunolide B | *p*-Cymene |
| 3,4-Dihydrocatalpol | Lactiflorin | Senkyunolide E | Senkyunolide A |
| Jioglutin D | Paeonianin A | Senkyunolide F | (*Z*)-Ligustilide |
| 2-Hydroxyaeginetic acid | Paeonianin B | Senkyunolide H | Senkyunolide I |
| 2,4-Dimethoxy-2-methyl-2H-pyran-3(6H)-one | Paeonianin C | Coniferyl ferulate | (*Z*)-Butylidenephthalide |
| Hierochin D | Paeonianin D | Sedanonic acid | (*E*)-Ligustilide |
| Glutinoside | Paeonianin E | Neocnidilide | Butylidenphthalide |
| Jionoside B1 | Debenzoylpaeonidanin | Butylphthalide | Butylphthalide |
| Jionoside D | Paeonin B | Butylidenephthalide | Senkyunolide E |
| Jionoside B2 | Paeonin A | Senkyunolide I | Senkyunolide F |
| Rehmapicrogenin | ε-Viniferin; (7*E*,7'*S*,8'*S*)-form | Senkyunolide K | Senkyunolide H |
| Rehmapicroside | ε-Viniferin; (7*Z*,7'*R*,8'*R*)-form | Senkyunolide L | Cnidilide |
| Jionoside E | Benzoic acid | Glucose | Levistolide A |
| Jionoside A1 | β-Glucogallin | Fructose | γ-Terpinene |
| Purpureaside C | Paeonol | Sucrose | Bergapten |
| Isoacteoside | Paeoniflorin | Scopoletin | Imperatorin |
| Jiofuran | (+)-Paeoniflorigenone | Pregnenolone | Falcarinol |
| Jioglutin E | Eugenin | Falcarindiol | Falcarinolone |
| Jioglutolide | 1,2,3,6-Tetra-*O*-galloyl-beta-D-glucose |  | Hydroferulic acid |
| Jioglutoside B | Oxypaeoniflorin |  |  |
| 8-Epiloganic acid | Paeonilactone A |  |  |
| Oxyrehmaionoside B | Paeonilactone B |  |  |
| Melasmoside | Paeonilactone C |  |  |
| Rehmaionoside B | Butanol |  |  |
| Rehmaionoside C | Catechin |  |  |
| Rehmaionoside A | Paeonolide |  |  |
| Rehmannioside D | β-Sitosterol |  |  |
| Glutinolic acid | Sucrose |  |  |
| Glutinosalactone A | Gallic acid |  |  |
| Jionoside C | 4-*O*-Methylgallic acid |  |  |
| Phenethyl primeveroside |  |  |  |
| Rehmaglutin A |  |  |  |
| Rehmaglutin D |  |  |  |
| Rehmaglutin B |  |  |  |
| Jioglutin A |  |  |  |
| Jioglutin B |  |  |  |
| Jioglutin C |  |  |  |
| Rehmaglutin C |  |  |  |
| Rehmaglutoside E |  |  |  |
| Rehmaglutoside J |  |  |  |
| Rehmaglutoside K |  |  |  |
| Rehmanone A |  |  |  |
| Rehmanone C |  |  |  |
| Rehmanone B |  |  |  |
| Catalpol |  |  |  |
| Melittoside |  |  |  |
| Aucubin |  |  |  |
| Dihydrocornin |  |  |  |
| Acteoside |  |  |  |
| Echinacoside |  |  |  |
| Stachyose |  |  |  |
| Raffinose |  |  |  |
| Sucrose |  |  |  |
| Manninotriose |  |  |  |
| Glucose |  |  |  |
| Cerebroside |  |  |  |

LC–HRMS, liquid chromatography–high-resolution mass spectrometry.

**Supplementary Table 3** Targeted LC–MS/MS methods: ion parameters of test ingredients of shimotsuto

| **Ingredient name** | **Q1 mass** | **Q3 mass** | **DP** | **CE** | **CXP** | **Method no.** |
| --- | --- | --- | --- | --- | --- | --- |
|  | *m/z* | *m/z* | volts | volts | volts |  |
| Catalpol | 379.918 | 183.100 | 51 | 9 | 22 | 1 |
| Paeoniflorin | 478.983 | 449.100 | −130 | −12 | −19 | 2 |
| Albiflorin | 479.007 | 121.100 | −125 | −22 | −9 | 2 |
| Ferulic acid | 192.954 | 133.800 | −10 | −22 | −15 | 2 |
| Senkyunolide A | 193.076 | 91.000 | 66 | 33 | 10 | 2 |
| Ligustilide | 191.100 | 115.000 | 86 | 25 | 10 | 3 |
| Butylphthalide | 191.050 | 145.100 | 41 | 19 | 8 | 3 |
| Bergapten | 217.215 | 202.000 | 86 | 29 | 10 | 4 |
| 8-Debenzoylpaeoniflorin | 375.240 | 194.900 | −110 | −20 | −19 | 5 |
| Swertiamarin (IS) | 375.100 | 195.200 | 60 | 15 | 14 | 1 |
| Imperatorin (IS) | 271.055 | 203.000 | 56 | 21 | 2 | 3 |
| Niflumic acid (IS) | 280.826 | 236.800 | −60 | −30 | −16 | 5 |
| Niflumic acid (IS) | 283.100 | 265.100 | 101 | 31 | 20 | 2, 4 |

DP, declustering potential; CE, collision energy; CXP, collision cell exit potential; IS, internal standard; LC–MS/MS, liquid chromatography–tandem mass spectrometry.

**Supplementary Table 4** Targeted LC–MS/MS methods: ion source parameters in LC–MS/MS analysis

| **Method no.** | **Target ingredient** | **CUR** | **IS** | **TEM** | **GS1** | **GS2** | **CAD** |
| --- | --- | --- | --- | --- | --- | --- | --- |
|  |  | **(psi)** | **(V)** | **(°C)** | **(psi)** | **(psi)** | **(psi)** |
| 1 | Catalpol | 30 | 5500 | 500 | 80 | 80 | 7 |
| 2 | Paeoniflorin  Albilforin  Ferulic acid | 30 | -4000 | 600 | 60 | 80 | 8 |
|  | Senkyunolide A | 10 | 4000 | 600 | 60 | 80 | 8 |
| 3 | Ligustilide  Butylphthalide | 10 | 4000 | 600 | 60 | 80 | 8 |
| 4 | Bergapten | 10 | 5000 | 600 | 50 | 80 | 12 |
| 5 | 8-Debenzoylpaeoniflorin | 30 | -4500 | 300 | 60 | 40 | 10 |

CUR, curtain gas; GS1, ion source gas 1; GS2, ion source gas 2; IS, ionspray voltage; TEM, temperature; CAD, collision-activated dissociation gas; LC–MS/MS, liquid chromatography–tandem mass spectrometry.

The method number corresponds to Supplementary Table 3.

**Supplementary Table 5** Targeted LC–MS/MS methods: HPLC conditions

| **Method no.** | **Measured ingredient** | **HPLC condition** |
| --- | --- | --- |
| 1 | Catalpol | Column: Atlantis dC18 column (100 × 2.1 mm I.D., 3.0-µm particle size; Waters)  Mobile phase: (A) 10 mM ammonium formate, (B) acetonitrile  Gradient elution program (% B): 0­–4 min, 5%; 4–10 min, 5%–95%; 10–15 min, 95%; 15.01–20 min, 5%  Flow rate, 0.20 mL/min; column temperature, 40°C |
| 2 | Paeoniflorin  Albilforin  Ferulic acid  Senkyunolide A | Column: Ascentis Express RP-amide column (100 × 2.1 mm I.D., 2.7-µm particle size; Supelco)  Mobile phase: (A) 0.2 vol % acetic acid, (B) acetonitrile containing 0.2 vol % acetic acid  Gradient elution program (% B): 0–2 min, 2%–5%; 2–22 min, 5%–60%; 22.01–27 min, 95%; 27.01–32 min, 2%  Flow rate, 0.30 mL/min; column temperature, 20°C |
| 3 | Ligustilide  Butylphthalide | Column: Ascentis Express RP-amide column (100 × 2.1 mm I.D., 2.7-µm particle size; Supelco)  Mobile phase: (A) ultrapure water, (B) acetonitrile  Gradient elution program (% B): 0–8 min, 40%–70%; 8.01–13 min, 95%; 13.01–18 min, 40%  Flow rate, 0.30 mL/min; column temperature, 20°C |
| 4 | Bergapten | Column: Atlantis dC18 column (100 × 2.1 mm I.D., 3.0-µm particle size; Waters)  Mobile phase: (A) 0.2 vol % acetic acid, (B) acetonitrile containing 0.2 vol % acetic acid  Gradient elution program (% B): 0–3 min, 50%–60%; 3–3.5 min, 60%–95%; 3.5–9 min, 95%; 9.01–14 min, 50%  Flow rate, 0.20 mL/min; column temperature, 20°C |
| 5 | 8-Debenzoyl-  paeoniflorin | Column: Acquity UPLC HSS T3 column (150 × 2.1 mm I.D., 1.8-µm particle size; Waters)  Mobile phase: (A) 0.2 vol % acetic acid, (B) acetonitrile containing 0.2 vol % acetic acid  Gradient elution program (% B): 0–5 min, 5%; 5–20 min, 5%–95%; 20–25 min, 95%; 25.01–30 min, 5%  Flow rate, 0.20 mL/min; column temperature, 20°C |

The method number corresponds to Supplementary Tables 3 and 4.

LC–MS/MS, liquid chromatography–tandem mass spectrometry; HPLC, high-performance liquid chromatography.

**Supplementary Table 6** Calibration curves used for quantification of shimotsuto ingredients

| **Ingredient** | **Range of quantification (ng/mL)** | **Correlation coefficient (*r*)** | **Range of accuracy (%)** |
| --- | --- | --- | --- |
| Catalpol | 2.00–2000 | 0.997 | 85.8–109 |
| Paeoniflorin | 1.00–200 | 0.995 | 86.8–107 |
| Albilforin | 0.500–50.0 | 0.999 | 94.9–106 |
| Ferulic acid | 0.500–100 | 0.997 | 90.7–108 |
| Senkyunolide A | 5.00–100 | 0.992 | 87.8–111 |
| Ligustilide | 1.00–50.0 | 1.000 | 99.9–100 |
| Butylphthalide | 1.00–50.0 | 0.995 | 92.5–109 |
| Bergapten | 0.0200–10.0 | 0.999 | 90.5–106 |
| 8-Debenzoylpaeoniflorin | 50.0–800 | 0.991 | 93.0–114 |

**Supplementary Table 7** HPLC conditions of nontargeted LC–HRMS

| **HPLC condition** |
| --- |
| Column: ACQUITY UPLC HSS T3 (2.1 × 150 mm, 1.8 μm; Waters) |
| Guard column: ACQUITY UPLC HSS T3 VanGuard Pre-column (2.1 mm × 5 mm, 1.8 µm; Waters) |
| Mobile phase (A) 0.1 vol % formic acid, (B) acetonitrile |
| Gradient elution program (% B):  0–24 min, 0.1%–99% |
| Flow rate, 0.4 mL/min; column temperature, 40°C |

LC–HRMS, liquid chromatography–high-resolution mass spectrometry; HPLC, high-performance liquid chromatography.

**Supplementary Table 8** Agonistic effect of shimotsuto ingredients against TRP channels

| **Crude drug** | **Test ingredient** | **% Activation compared to positive control against each subfamily of TRP channel** | | | | **EC_50_ against TRPA1 (µmol/L)** |
| --- | --- | --- | --- | --- | --- | --- |
|  |  | **TRPA1** | **TRPV1** | **TRPV4** | **TRPM8** |  |
| Rehmanniae Radix | Catalpol | 2 | 0 | 0 | 3 | － |
| Paeoniae Radix | 8-Debenzoylpaeoniflorin | 30 | 1 | 1 | 2 | － |
|  | Paeoniflorin | 1 | −1 | 0 | 0 | － |
|  | Albiflorin | 1 | 0 | −1 | 1 | － |
| Angelicae Acutilobae Radix or Cnidii Rhizome | Senkyunolide A | 86 | 1 | 0 | −1 | 1.0 |
|  | Ligustilide | 87 | 0 | -1 | 18 | 1.3 |
|  | Butylphthalide | 73 | −1 | 0 | −1 | 3.2 |
|  | Ferulic acid | 3 | 0 | 0 | 5 | － |
|  | Bergapten | 3 | 0 | 0 | 0 | － |

－, Not determined.

Calcium ion influx into TRPA1-, TRPV1-, TRPV4-, or TRPM8-expressing T-REx-293 cells was measured to evaluate the agonistic effect of shimotsuto ingredients on TRP channels. The action of 10 µmol/L of each test ingredient was represented as the relative enhancement of the Ca^2+^ influx of each positive control, indicated as follows: 20 µmol/L each of allyl isothiocyanate (TRPA1 agonist), 2 µmol/L capsaicin (TRPV1 agonist), 4 µmol/L GSK1016790A (TRPV4 agonist), and 20 µmol/L icilin (TRPM8 agonist). Dose–response tests of shimotsuto ingredients against TRPA1 were performed to calculate EC_50_.

TRP, transient receptor potential; TRPA1, transient receptor potential ankyrin 1; TRPV1, transient receptor potential cation channel subfamily V member 1; TRPV4, transient receptor potential cation channel subfamily V member 4; TRPM8, transient receptor potential cation channel subfamily M member 8; EC_50_, half-maximal effective concentration.
